# Long Island College Hospital. Cases of Paralysis Connected with Dentition

**Published:** 1860-07

**Authors:** F. H. Hamilton


### ARTICLE XVII.

*Long Island College Hospital. Cases of Paralysis connected with Dentition; with Remarks by* PROF. F. H. HAMILTON.

THERE have been recently presented to the class six cases of paralysis connected with dentition.

*Case* 1.—A little girl nineteen months of age, in whose case the left arm is paralysed. There is wasting of the deltoid and of the muscles of the arm and forearm—with complete loss of power. The paralysis is of three months standing.

*Case* 2.—A girl of three years of age—paralysis of the left leg. The heel is raised considerably from the ground. The muscles of the leg are more wasted than those of the thigh. In attempting to walk, she drags the foot sideways.

*Case* 3.—Mary Gorham, two years and one month old. Eight months ago she had a fever, after this the mother noticed that the child could not use the left leg. There is a tendency of the foot to rotate outward, she drags the toe. Paralysis is mostly from the knee downwards. There is wasting of the limb. In this case it is to be attributed to the fever. Prof. Hamilton has never seen a case in which infantile paralysis could be attributed to the abuse or use of calomel.

*Case* 4.—Boy three years and a half old, in all respects healthy, except loss of power of left leg. No traceable cause, unless diarrhea attending teething. The limb is wasted and heel is elevated, there is dragging of the toe—sensation in the limb is perfect.

*Cases* 5 *and* 6.—Were both boys, and the paralysis confined to the right leg—presenting the same symptoms of wasting of the limb, loss of power, elevated heel, &c.; no assignable cause for the paralysis.

*Remarks.*—Infantile paralysis occurs generally at some time during the period of first teething, and seems to have a more or less direct connection with this process. There are several periods in life, in which the system or portions of the system undergo remarkable changes, and great nervous derangements are apt to ensue. The period of the first dentition, is one of these, and among the consequences of the nervous disturbances occasioned by the development of the teeth, are convulsions, hydrocephalus, diarrhea, and *paralysis*. The other periods to which I allude, are puberty in both male and female, and "the change of life," or the period of cessation of the menses in the female. I think that I have observed, also, that at the period of "change of life" in the male, paralysis and cerebral apoplexy are exceedingly liable to occur.

Infantile paralysis is more common in the legs than in the arms; yet we see it occasionally in the arms. It generally exists but in one leg, occurring in most cases suddenly, and not unfrequently after a diarrhea or fever of some form, or after exposure to the cold. Its frequent sequence to some temporary illness has often led parents to suppose that it was due to the medicine which had been given, especially to mercury; but I have never been able to trace it to the use of mercury, in any form. I have never seen the paralysis complete; it being, in the case of the lower extremities, confined mostly to that portion of the limb which is below the knee, and existing much more in the nerves of motion than in those of sensation.

The child can generally stand upon, and even walk with the limb tolerably well; but the foot turns out whenever the weight of the body rests upon it, and the gait is unsteady and insecure. He is especially unable, when sitting, to raise and extend the leg upon the thigh, but he can generally flex the thigh upon the body without difficulty. After a time the muscles become sensibly diminished in size, especially the muscles of the leg; and if the paralysis continues, the leg does not increase in length quite as rapidly as the other, and the foot becomes more and more turned out at the ankle.

*Treatment.*—First of all, if the case is seen soon after the occurrence, the bowels should be well evacuated, and the condition of the gums should be looked to. The child should then be turned out of doors, and be permitted to gather health and strength by exercise in the open air. By use, mainly, are these muscles to be again restored to action. The little patient should therefore be encouraged to walk, and if old enough he must be directed to sit occasionally upon a chair and swing his leg back and forward in flexion and extension. No apparatus ought to be employed to support the ankle or the knee, unless these joints are becoming greatly deflected, or the limb is totally unable to support its weight. When apparatus is substituted for the muscles, the muscles being thrown into disuse, are not in a condition to gain strength. Upon this point there is a popular error which needs to be corrected. When a young man begins to stoop, or his spine begins to fall to one side or the other, his friends tell him he must get a shoulder-brace or a spine-supporter—and there are always plenty of them hanging in the windows of drug-shops, patented and recommended for this very purpose. Now the probability is, that this fall of the shoulders or curvature of the spine, is entirely due to muscular weakness. The great trapezoid does not pull the scapula well back, and if we pull them back with a shoulder-brace, leaving the muscles nothing to do, they will rapidly become atrophied, and when the brace is taken off the shoulders will fall forward more than ever. It would be much better to put the brace in front, and tie the shoulders forward, and then by the constant antagonism the trapezoid would be strengthened. Those persons who carry weights upon their heads, grow straight; the spine being made to erect itself by the action of the muscles, whose power is thus developed and perfected. It is this use of the muscles in carrying the gun, which makes the soldier and the hunter so erect. The same principle applies to these cases of infantile paralysis. The limb should be kept in constant use, and no apparatus should be employed to support the limb, except in the few extreme cases mentioned. It is well, also, to bathe the limbs in cold water once a day, and to rub them freely afterwards. Galvanism and electricity also deserve a trial.

Usually the recovery is very slow, extending through a period of several years; but I have been enabled to follow the history of enough of these cases to warrant me in assuring the parents that they will almost certainly improve, and that there is a reasonable probability of a final and complete recovery.—*Am. Med. Times.*

---

